# Rescue ALPPS is efficient and safe after failed portal vein occlusion in patients with colorectal liver metastases

**DOI:** 10.1007/s00423-016-1524-y

**Published:** 2016-10-19

**Authors:** Ernesto Sparrelid, Stefan Gilg, Torkel B. Brismar, Lars Lundell, Bengt Isaksson

**Affiliations:** 1 Division of Surgery, Department of Clinical Science, Intervention, and Technology (CLINTEC), Center for Digestive Diseases, Karolinska University Hospital, Karolinska Institutet, Stockholm, Sweden; 2 Department of Surgery, Centre for Digestive Diseases, Karolinska University Hospital, Stockholm, Sweden; 3 Division of Radiology, Department of Clinical Science, Intervention, and Technology (CLINTEC), Karolinska University Hospital, Karolinska Institutet, Stockholm, Sweden

**Keywords:** Liver resection, Colorectal liver metastases, ALPPS, PVO

## Abstract

**Purpose:**

The aim of this study was to investigate whether associating liver partition and portal vein ligation for staged hepatectomy (ALPPS) can be used as an effective and safe rescue procedure in patients with colorectal liver metastases (CRLM) and insufficient effect on the future liver remnant (FLR) after previous portal vein occlusion (PVO).

**Methods:**

Eleven patients with bilobar CRLM treated with neoadjuvant chemotherapy and previous PVO with insufficient effect on the FLR were analyzed retrospectively from a prospective database. FLR was evaluated with computed tomography volumetry 6 days after stage 1, and stage 2 was performed on day seven.

**Results:**

Six days after stage 1, the median increase of the FLR was 209 ml (range 87–314, *P* < 0.001). This corresponded to a median FLR growth of 61.8 % (range 19.3–120) resulting in an FLR/BW ratio >0.5 % in all patients and successful subsequent removal of the tumor bearing liver (segments IV–VIII) in all patients with no 90-day mortality. No patient had a 3b-complication or more according to Clavien-Dindo. No patient developed severe posthepatectomy liver failure.

**Conclusions:**

The powerful hypertrophy of the FLR associated with ALPPS seems to be maintained in patients with CRLM and previous failed PVO.

## Introduction

Portal vein occlusion (PVO) by either selective embolization (PVE) or ligation (PVL) of the portal vein to the tumor bearing part of the liver is an established method to increase the size of the future liver remnant (FLR) [1, 2]. The main purpose of this procedure is to convert previously unresectable patients to resection candidates by achieving sufficient size of the FLR before hepatectomy, in order to avoid posthepatectomy liver failure (PHLF) [3].

However, it has to be recalled that about one third of the patients submitted to PVO eventually never undergo a curative resection due to either insufficient growth of the FLR with an unacceptable risk of PHLF if submitted to surgery, or they progress to an unresectable local tumor situation while awaiting the full PVO effect [4, 5].

Associating liver partition and portal vein ligation for staged hepatectomy (ALPPS) has rendered great attention since its introduction [6, 7]. Although showing promising results with unprecedented growth of FLR, both in time and size, the technique has also been subjected to criticism, mainly because of high morbidity and mortality rates [8, 9]. A recent review of the literature shows that the majority of the serious complications affect patients subjected to concomitant biliary surgery [6, 10, 11], while patients with colorectal liver metastases (CRLM) undergoing ALPPS seem to be less prone to develop high-grade complications [11]. In an attempt to address patients with insufficient growth of the FLR after PVO, a modified ALPPS with only parenchymal transection (“rescue” ALPPS) has been reported in a few small case series [12–15].

The aim of this study was to investigate whether ALPPS can be used as an effective and safe rescue procedure in patients with CRLM and insufficient effect on the FLR after previous PVO.

## Material and methods

### Patients

From November 2012 to June 2015, 11 patients were included in the study from a prospective database at the Center for Digestive Diseases at Karolinska University Hospital. All patients presented with bilobar CRLM and had been treated with neoadjuvant chemotherapy. The patients were previously subjected to PVO (PVE, PVL, or both) with insufficient effect on the FLR and subsequently operated with rescue ALPPS. All patients were discussed at the local MDT conference. A ratio between FLR and body weight (FLR/BW ratio) of less than 0.5 % was considered as an indication for ALPPS [16]. Six days after stage 1, FLR was evaluated with computed tomography (CT) volumetry and stage 2 was performed on day seven. All patients had bilobar tumor manifestation but only four had metastases in segment I, II, or III requiring tumor clearance of the FLR at stage 1 (the remaining had metastases in segment IV in addition to the tumors in the right liver lobe). All patients were treated with neoadjuvant chemotherapy with a median of seven cycles (range 4–14). The treatment agents consisted of FOLFOX or FOLFIRI +/− a biological agent. The time elapsed from completion of chemotherapy treatment to stage 1 operation in the study was in median 85 days (range 26–172), as a result of the waiting time from PVO to ALPPS given the fact that chemotherapy was not given after PVO. The clinicopathological characteristics of the patient cohort are presented in detail in Table 1.

**Table 1:** Clinicopathological characteristics of the patient cohort (*n* = 11) before ALPPS

Variable	Rescue ALPPS (*n* = 11)
Median age, years (range)	67 (41–74)
Male/female gender	8/3
Median BMI (range)	26.1 (22.2–29.4)
ASA-class 1–2	9
ASA-class 3	2
Synchronous/metachronous metastases	6/5
Median number of liver metastases (range)	7 (2–20)
Tumor localization	
Right lobe + segment 4	7
Right lobe + segment 4 + left lateral segment	4
Chemotherapy before ALPPS	
Oxaliplatin based	7
Irinotecan based	4
Targeted therapy	5
Number of chemotherapy cycles (range)	7 (4–14)
Days between chemo and ALPPS (range)	85 (26–172)
Portal vein occlusion prior to ALPPS	
Portal vein embolization (PVE)	5
Portal vein ligation (PVL)	4
First PVL then PVE	2
TELV, ml (range)	1651 (1436–2070)
FLR before PVO, ml (range)	250 (180–370)
FLR/BW ratio before PVO, % (range)	0.33 (0.25–0.40)
sFLR before PVO, % (range)	14.9 (11.9–19.7)
Growth of FLR after PVO, % (range)	26.8 (−7.3–66.7)
Days from PVO to CT	28 (19–33)
FLR before ALPPS, ml (range)	312 (260–450)
FLR/BW ratio before ALPPS, % (range)	0.41 (0.35–0.49)
sFLR before ALPPS, % (range)	18.7 (16.1–23.8)

*ASA* American Society of Anesthesiologists Physical Classification System, *TELV* total estimated liver volume, *FLR* future liver remnant, *sFLR* standardized FLR, *FLR/BW ratio* FLR to body weight ratio

### Surgical technique

PVE was performed with percutaneous ipsilateral technique and puncture of peripheral portal branches of the right side. Polyvinyl alcohol beads (Terumo Bead Block™ Embolic Bead, Metron Healthcare, Athens, Greece) and polyvinyl alcohol particles (Contour™, Boston Scientic, Cork, Ireland) were combined with central coils (MicroNester® Embolization Coil, Cook, Indianapolis, USA), placed in the right portal vein, to obtain occlusion of the portovenous system to segment V–VIII. The portal branches to segment IV were not embolized as this is not routine at our center. PVL was performed by division of the right portal vein using a stapler instrument (Endo GIA™ Universal with Tri-Staple™, Covidien, Dublin, Ireland). PVL was preferred over PVE only when the FLR contained metastases and used together with local resections in FLR in the stage 1 operation of an intended conventional two-stage hepatectomy. ALPPS was performed in a similar way as described previously [6] in the patients with failed PVE. In summary, stage 1 comprised of division of the right portal vein as in PVL and complete parenchymal transection to the right of the falciform ligament using cavitron ultrasonic surgical aspirator (CUSA®, Valleylab Inc., Boulder, CO, USA). In rescue ALPPS after failed PVL, only parenchymal transection was performed at stage 1. At stage 2, performed 7 days later in all patients, the right portal pedicle and right liver vein were divided using the same stapler instrument as above and the tumor bearing deportalized liver could be removed as in an extended right-sided hemihepatectomy preserving only segment I–III in all patients.

### Volumetric analysis

**Fig. 1:**
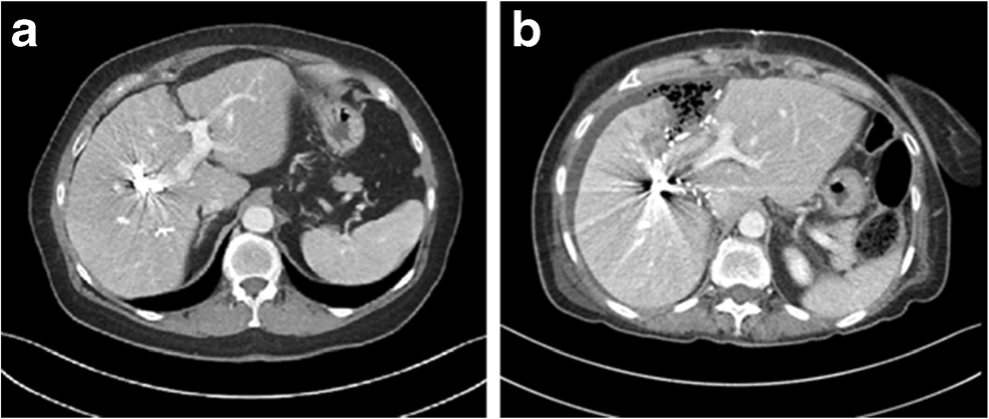
Example of rescue ALPPS after failed portal vein embolization CT after PVE (**a**) and CT before stage 2 (**b**) in the same patient

Liver volume was calculated from a four-phase contrast enhanced CT of the liver by using the software Volume Viewer© (Voxtool 11.x) for AW Volume Share 5 implemented on an AW Workstation (GE Healthcare, Fairfield, CT, USA). The CT evaluating the effect of the PVO was performed after 28 days in median (range 19–33) and was used as baseline investigation (Table 1). To measure the effect on the FLR, all patients underwent a second CT of the liver on day six after stage 1 (Fig. 1). The FLR/BW ratio was calculated and total estimated liver volume (TELV) was calculated according to the formula developed by Vauthey and co-workers to obtain standardized FLR (sFLR) [17].

### Data collection and statistical analysis

Baseline patient characteristics, volumetric data, procedural data, and complications were collected prospectively in a local database. Statistical analysis was performed using JMP® version 5.1 (SAS Institute Inc., Cary, NC, USA). Median values (range) were used for continuous variables whereas frequencies were calculated for categorical variables. Paired *t* test was used to compare means between different time points in the same patient. *P* values of <0.05 were considered to represent statistical significance.

The study protocol was approved by the Central Ethical Review Board, Stockholm, Sweden.

## Results

### Clinical outcome

The median operating time for stage 1 was 282 min (range 200–398) with an intraoperative blood loss of 1500 ml (range 400–5600). In the four patients requiring tumor clearance of the FLR, local resections were performed in two patients, and in two patients, a combination of local resections and microwave ablation (MWA) was used. All these procedures for metastases in the FLR were undertaken during the stage 1 procedure. All patients could complete stage 2 with the removal of the tumor bearing deportalized liver. Radical resection (R0) was achieved in all 11 patients. The complication rate was low. Four patients had pleural effusion that was drained in local anesthesia, consequently recorded as a 3a-complication according to the Clavien-Dindo classification [18, 19]. In one patient, this occurred after stage 1 and in the remaining three after stage 2. There were no complications equal to or above 3b. No patient fulfilled the Balzan 50/50 [20], peak bilirubin >7 [21], or ISGLS [22] criteria grade C for severe postoperative liver failure and there was no 90-day mortality. Detailed postoperative clinical data are shown in Table 2.

**Table 2:** Procedural, clinical, and volumetrical data after completed ALPPS

Variable	Rescue ALPPS (*n* = 11)
Operating time stage 1, min (range)	282 (200–398)
Bleeding during stage 1, ml (range)	1500 (400–5600)
Completed stage 2	11
R0-resection	11
Complication (Clavien-Dindo) 3a	4
Complication (Clavien-Dindo) ≥3b	0
Fulfills Balzan criteria	0
90-day mortality	0
FLR after stage 2, ml (range)	557 (395–619)
FLR/BW ratio before stage 2, % (range)	0.69 (0.59–0.81)
sFLR before stage 2, % (range)	31.2 (27.5–36)
Growth of FLR between stage 1 and 2, % (range)	61.8 (19.3–120)
PK (INR) 1 day before stage 1	1.0 (0.9–1.1)
PK (INR) 5 days after stage 1	1.2 (1.1–1.5)
PK (INR) 5 days after stage 2	1.3 (1.2–1.8)
Bilirubin 1 day before stage 1	6 (3–14)
Bilirubin 5 days after stage 1	8 (6–22)
Bilirubin 5 days after stage 2	17 (8–49)

*R0-resection* radical resection with >1 mm margin, *PK (INR)* normal if <1.2, *Bilirubin* normal if <26 micromol/l

### Growth of the FLR

**Fig. 2:**
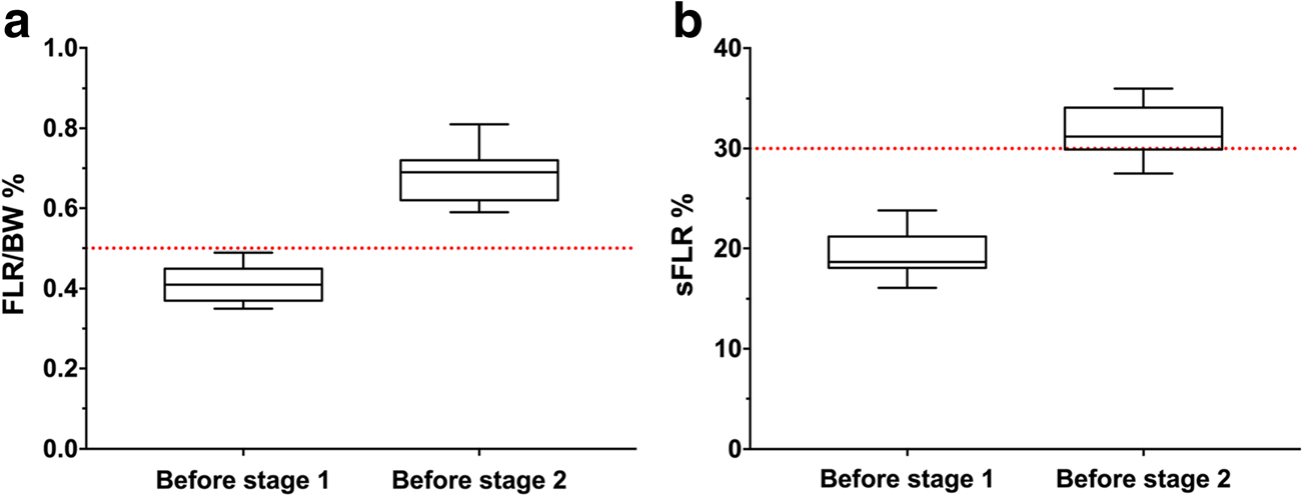
Box-whisker plot displaying the increase in FLR/BW ratio (**a**) and sFLR (**b**) between stage 1 and 2 of ALPPS in patients subjected to rescue ALPPS. **a** Increase in FLR/BW ratio. *Dotted red line* shows 0.5 %. **b** Increase in sFLR. *Dotted red line* shows 30 %

FLR volume before PVO was 250 ml (range 180–370). After PVO the FLR increased to 312 ml (range 260–450), representing a growth of the FLR with 26.8 % (range − 7.3-66.7, *P* = 0.006). All patients responded well to the ALPPS stage 1 procedure despite previous PVO. Six days after stage 1, the median increase of the FLR was 209 ml (range 87–314, *P* < 0.001). This corresponded to an increase of FLR/BW ratio to 0.69 % (range 0.59–0.81), i.e., reaching more than 0.5 % in all patients (Fig. 2a). In Fig. 2b, the corresponding increase in sFLR is also presented. The growth of the FLR between stage 1 and 2 was in median 61.8 % (range 19.3–120). For further details regarding volumetrical data, see Tables 1 and 2.

## Discussion

This study confirms results from previous smaller case reports that ALPPS can be both safe and effective as a rescue procedure in CRLM patients with insufficient effect on the FLR after previous PVO. In addition, it seems as if the previously described high morbidity and mortality associated with ALPPS does not apply when performing the procedure in this patient group.

As mentioned, rescue ALPPS for CRLM has been described previously [12–15], although in only quite few patients. Despite the limited number of patients enrolled, the patient cohort of this study still represents the largest series of rescue ALPPS for CRLM so far presented, and in addition, the main aim of this study was to specifically investigate rescue ALPPS. In one recent study [15], nine patients with ALPPS after PVE were reported but without stating the background diagnosis and chemotherapy regimens in those subjected to PVE before ALPPS. Some of the patients in the present study were also included in a Scandinavian multicenter study recently published [23]. However, this study also reported a mixed study population and did not have the aim to study rescue ALPPS. To analyze the effect and consequences of a complex procedure such as ALPPS, we considered it as an advantage to have a patient population as homogenous as possible to minimize the effects of a variety of confounding factors. Accordingly, we only included patients with bilobar CRLM; all of them subjected to neoadjuvant chemotherapy and with an FLR consisting of segment I–III in all patients.

In the present study cohort, the overall median growth of the FLR before stage 2 was 61.8 %, which is less than reported in most previous studies. This might, however, be explained by two factors. Firstly, in contrast to the majority of other reports on ALPPS, only patients with CRLM treated with pre-procedural chemotherapy were included. It is recognized that pre-PVE chemotherapy can have a negative effect on growth of the FLR after PVE [24, 25] and it is likely to have the same effect after ALPPS, although this remains to be proven. Another complicating factor was that the interval between the cessation of chemotherapy and ALPPS was quite long due to the waiting time from PVO to ALPPS. Consequently, the alleged negative effect of pre-procedural chemotherapy on FLR hypertrophy might have been less pronounced in these patients compared to the patients undergoing ALPPS without previous PVO. Secondly, the inter-stage time of 7 days used for all patients in this study was shorter than in the most published series [26]. That might in part explain the somewhat less pronounced hypertrophy rate. Also, the volume increase of the FLR seems to be paired with a functional increase since no patient developed severe liver failure after stage 2. This absence of severe PHLF was seen despite the fact that a FLR/BW ratio below 0.5 % was used for inclusion, compared to other centers that use a FLR/BW ratio below 0.8 % or a sFLR below 30 % for deciding to perform ALPPS in patients with pre-procedural chemotherapy [27, 28]. In a recent retrospective study at our institution comparing different volumetric methods for characterizing the size of the FLR before and after PVE, an FLR/BW ratio of 0.5 % corresponded to a sFLR of 22.9 % and thus clearly below sFLR of 30 % (unpublished data).

The observed low morbidity and zero mortality are probably attributed to the inclusion of only patients with CRLM as mentioned above. In the first publication from the ALPPS Registry, age above 60 years was reported as a risk factor for mortality after ALPPS in CRLM [11]. The median age in this study population was 67 years, but we speculate that our low patient co-morbidity might have contributed to the absence of 90-day mortality. The resection rate in this study was 100 %, similar or higher to what has been achieved in previous studies, which clearly surpasses the resection rate after PVO and two-stage hepatectomy [29]. A potential confounder might be that some patients subjected to PVO will experience tumor progression while waiting for the effect of the PVO excluding them from subsequent surgery, thus leading to a possible selection bias with a more favorable tumor situation for patients undergoing rescue ALPPS compared to those submitted directly to standard ALPPS.

The question whether ALPPS should be used more frequently in patients with CRLM and a small FLR remains to be answered. In this study, ALPPS was used successfully as a rescue method after failed PVO. Considering the potent growth of the FLR after ALPPS, patients with CRLM and very small FLR and/or monosegment FLR should probably be considered for ALPPS upfront [30].

## Conclusion

The powerful FLR growth associated with ALPPS seems to remain even when performed in patients with previous PVO. Apparently, in patients with colorectal liver metastases subjected to previous PVO, ALPPS can be performed with low morbidity and high resection rate.
